# Study on the effect of GCY-12 gene on albendazole sensitivity of *Haemonchus contortus* by RNA interference

**DOI:** 10.3389/fvets.2025.1567869

**Published:** 2025-05-12

**Authors:** Xindi Chen, Yaqin Shi, Tengyu Wang, Chunxia Liu, Wenlong Wang, Yu Wang

**Affiliations:** ^1^Key Laboratory of Animal Disease Clinical Diagnosis and Treatment Technology, College of Veterinary Medicine, Inner Mongolia Agricultural University, Hohhot, China; ^2^Key Laboratory of Animal Disease Clinical Diagnosis and Treatment Technology, College of Life Science, Inner Mongolia Agricultural University, Hohhot, China

**Keywords:** *Haemonchus contortus*, albendazole, drug resistance, GCY-12, RNA interference

## Abstract

**Introduction:**

*Haemonchus contortus* (*H. contortus*) is a common gastrointestinal nematode in small ruminants, posing a significant threat to the livestock industry. The control of *H. contortus* often depends on drugs such as albendazole. However, the prolonged and improper use of these drugs by livestock producers has led to widespread resistance among ruminant populations, posing a major challenge to parasite management. It is reported that the resistance of *H. contortus* to albendazole is related to the single nucleotide polymorphism (SNP) of β-tubulin homologous type I gene, but whether other genes are involved has not been reported.

**Methods:**

Based on the comparative analysis of the transcriptome sequencing data of albendazole-sensitive and albendazole-resistant strains of *H. contortus*, HCON_00043720 (Receptor-type guanylate cyclase GCY-12, GCY-12) gene was selected as the research object from the 23 differential genes in the cyclic guanosine monophosphate (cGMP) signaling pathway where the growth and development of *H. contortus* dauer stage is located. The GCY-12 gene of *H. contortus* eggs was silenced by RNA interference (RNAi) test. The reaction temperature was optimized, the expression of silenced eggs was detected by quantitative real-time polymerase chain reaction (qRT-PCR) technology, and the drug resistance of silenced eggs was detected by egg hatch assay (EHA).

**Results:**

The results showed that the gene expression level decreased significantly after GCY-12 interference, and the sensitivity of *H. contortus* to albendazole increased.

**Discussion:**

This study highlights the potential role of GCY-12 in modulating albendazole resistance in H. contortus, offering new insights for developing effective therapeutic approaches.

## Introduction

*Haemonchus contortus* exists in the abomasum of small ruminants (1). The primary clinical manifestations of *H. contortus* infection include progressive weight loss, visual mucosal pallor, and mandibular edema. In severe cases, the host may eventually die due to extreme weakness and malnutrition (2, 3). *H. contortus* has exhibits strong adaptability to diverse environments, and its high genetic polymorphism may confer selective advantages by enhancing environmental tolerance. Consequently, parasitic nematodes, represented by *H. contortus* (4), are prevalent in nearly all regions where ruminants are raised and have been extensively reported worldwide (2, 5–10).

Currently, gastrointestinal nematode diseases represented by *H. contortus* are mainly prevented and treated with anthelmintic drugs, and some animal and plant extracts and compounds also have certain anthelmintic therapeutic effects (11–13). The common deworming drugs are albendazole and ivermectin (14), which have good deworming effect in clinic, but with the long-term use of deworming drugs in the same area, especially unreasonable application, some parasitic nematodes represented by *H. contortus* have gradually developed resistance to common deworming drugs (15–20). The effects of albendazole on *H. contortus* were again affected by reproduction and environmental circulation of the resistant worms (21). Albendazole is a broad-spectrum benzimidazole anthelmintic. It mainly inhibits microtubule polymerization by binding to β-tubulin, affecting mitosis and protein assembly of nematode worms. This mechanism impairs various cellular processes, including nutrient absorption, intracellular transportation and energy metabolism, ultimately leading to parasite paralysis, hunger and death (22, 23). Current research indicates that albendazole resistance primarily arises from SNP in the type I β-tubulin gene of *H. contortus*, specifically at positions 167 (F167Y) and 198 (E198A), and 200 (F200Y) (24–26), which significantly reduce β-tubulin's binding affinity. The increased expression and activity of some metabolic enzymes may be one of the mechanisms of nematode resistance to benzimidazole anthelmintics, such as cytochrome P450 enzymes. In addition, some studies have shown that adults have a strong level of drug metabolism, so the increase in the efficiency of drug metabolism may also be one of the factors of nematode drug resistance (27, 28).

According to the diapause larvae hypothesis in the model organism *Caenorhabditis elegans* (*C. elegans*), exposure to adverse environmental conditions triggers the transition from the late second stage larvae (L2) to dauer stage (29, 30). In this state, larvae undergo metabolic and physiological changes that induce “dormancy,” characterized by a closed mouth and an outer protective sheath, allowing them to withstand harsh environments. Once conditions become favorable, larvae resume development into fourth stage larva (L4). During the dauer stage, nematodes cease growth and reproduction, exhibit significantly reduced metabolic activity, and demonstrate high resistance to environmental stressors. The infectious third-stage larvae (L3) of *H. contortus* are significantly similar to the dauer stage larvae of *C. elegans*. Given the extensive research on *C. elegans* as a model organism, we hypothesize that *H. contortus* may employ similar mechanisms to develop drug resistance, such as reducing metabolic rates, modifying drug absorption pathways, or enhancing drug tolerance through specific genetic regulation. Four important signaling pathways are involved in dauer stage: cGMP signaling pathway, insulin/insulin-like signaling pathway (ILS), transforming growth factor β signaling pathway (TGF-β) and steroid hormone biosynthesis signaling pathway (31–34). Currently, no studies have examined dauer pathway-related genes in the context of drug resistance in *H. contortus*. In addition, studies have shown that P-glycoprotein (P-gp), as a type of ATP-dependent membrane transport and efflux pump, is involved in typical physiological detoxification and host defense activities (35). Overexpression of P-gp in tumor cells is a major contributor to multidrug resistance (MDR) by actively exporting drugs and reducing intracellular drug concentrations, thereby diminishing the efficacy of chemotherapy. Based on the above views, in order to explore whether the over-expressed genes in the response pathway of *H. contortus* to harsh environments will further affect the changes in drug resistance, this study based on the transcriptome sequencing analysis of previous albendazole-sensitive and albendazole-resistant strains of *H. contortus*, identified the gene GCY-12, which has the highest proportion of different genes in the four pathways and is significantly enriched in the cGMP signaling pathway (belonging to the receptor-type guanylate cyclase family, it can catalyze the conversion of guanylate to cyclic guanylate, thereby activating downstream cGMP dependent signaling pathway and participating in the regulation of dauer stage development and regulation related to exercise behavior). Moreover, GCY-12 was also involved in the enrichment of nine signaling pathways including cAMP signaling pathway and renin secretion signaling pathway, suggesting that it may have a critical role in multiple biological processes. Therefore, further investigation into the role of GCY-12 in *H. contortus* drug resistance is crucial for elucidating its potential regulatory function anthelmintic resistance mechanisms. To this end, we conducted RNAi and qRT-PCR detection on the eggs of *H. contortus* and verified the gene resistance correlation, revealing the biological function of the GCY-12 gene. The research results can provide a theoretical basis for further exploring the mechanism of albendazole resistance and the development of novel anthelmintic.

## Materials and methods

### Collection of eggs and L3 of *H. contortus*

Eight months old sheep were transported from a pasture to the College of Veterinary Medicine, Inner Mongolia Agricultural University, for captive rearing. The sheep were treated with closantel sodium, and fecal samples were collected 5 days post-treatment for parasite detection using the McMaster technique. Twenty days post-treatment, when the mean fecal egg count (FEC) reached zero, the sheep were experimentally infected with L3 of albendazole-resistant *H. contortus* preserved in the laboratory. These larvae were originally isolated from sheep feces in Ulanhot, Inner Mongolia, with an initial host mean FEC of 3,600 eggs per gram (EPG). The resistant strain was characterized by a fecal egg count reduction (FECR) of 13.8% and a half effect concentration (EC_50_) of 1.28 μg/mL, as determined by fecal egg reduction test (FECRT) and EHA. Each sheep was infected with 8,000 L3s. Thirty days post-infection, *H. contortus* specimens were collected.

Acquisition of *H. contortus* Eggs: fresh feces samples were collected directly from the rectum of sheep confirmed to be infected with albendazole-resistant strains, and eggs were immediately isolated. A 20 g fecal sample was placed in a beaker, mixed thoroughly with an appropriate volume of saturated saline solution, and sequentially filtered through 0.27 mm, 0.075 mm, and 0.048 mm sieves. The filtrate, free of fecal debris, was then mixed with a sucrose solution (saline solution: sucrose solution = 4:1), stirred evenly, and transferred into a plate, A thin plastic sheet was carefully placed on the liquid surface to ensure full contact without overflow. After 30 min of sedimentation, the plastic sheet was gently removed, rinsed with distilled water, and the rinse solution was filtered through a 0.025 mm sieve. The collected eggs were washed with sterile, enzyme-free water and subjected to three rounds of centrifugation at 1,000 × g for 2 min each.

Acquisition of *H. contortus* L3: collected fecal samples were thoroughly homogenized and placed in a plastic container, covered with a layer of moist gauze, and incubated at 27°C in a biochemical incubator for 7 days. Water was regularly sprayed to maintain moisture, and the samples were periodically stirred to provide oxygen for larval development and prevent mold formation. After 7 days, a small portion of the fecal culture was smeared onto a microscope slide, excess debris was removed, and the vitality and morphology of the larvae were examined under the microscope. Once confirmed that *H. contortus* had developed into the L3 stage, larvae were collected using the Behrman technique (36). The fresh L3 were washed three times and examined under microscope before being reserved for further experiments.

Acquisition of exsheathed third-stage larvae (xL3) of *H. contortus* (11): Fresh *H. contortus* L3 were treated with 1% sodium hypochlorite solution and incubated in a 37°C water bath for 20 min. The L3 were then washed three times with RNase-free water before further processing.

### Differential expression genes of signaling pathways related to dauer stage

Based on the results of the transcriptional groups of albendazole-sensitive and albendazole-resistant strains of *H. contortus* measured earlier by our research team, the differential genes enriched in cGMP signaling pathway, ILS signaling pathway, TGF-β signaling pathway and hormone-like signaling pathway were selected as candidate genes with the threshold of |log_2_Foldchange| ≥ 1 and FDR < 0.05.

### Silencing of the GCY-12 gene

In *C. elegans*, the GCY gene is closely related to the function of nematode sensing environmental cues such as chemical signals, oxygen and temperature (37, 38). The GCY gene family encodes guanylate cyclase proteins, which are involved in the production of the second messenger cGMP. The cGMP signaling pathway regulates the perception of environmental stimuli and neuronal functions in nematodes (39). To investigate the potential role of the GCY gene in *H. contortus* resistance to albendazole, this study focused on GCY-12, a differentially expressed gene identified within the cGMP signaling pathway. Transcriptome sequencing revealed that the coding sequence (CDS) of *H. contortus* GCY-12 is 1,059 bp in length. Small interfering RNA (siRNA) targeting GCY-12 and a nonspecific negative control siRNA (NC siRNA) were designed and synthesized by Shanghai GenePharma Co. Ltd (40–42). The siRNA design followed standard principles: target sequences were primarily selected within the CDS region, sense and antisense strands were 19–21 nucleotides in length, and GC content ranged from 30% to 60%. Sequences with more than four consecutive adenines (A) or thymidines (T) were avoided, and target sequences were evenly distributed. Homology analysis was conducted using BLAST in the National Center for Biotechnology Information (NCBI) database, and sequences with <78% homology to *H. contortus* were selected (only the sequences were checked for homology to the *H. contortus* genome). Three siRNAs were designed based on these criteria, and those demonstrating effective gene silencing in preliminary experiments were used for further analysis. The NC siRNA, a universal control sequence with no homology to *H. contortus*, has been widely reported in the literature (43) (Table 1). The fresh xL3 of *H. contortus* were washed with RPMI 1640 medium containing penicillin-streptomycin and amphotericin B, and impurities were removed. The L3 were then transferred to 24-well cell culture plates for RNAi experiments. The experimental setup included the following groups: the siRNA group (targeted interference), the NC group (negative control transfected with non-targeted sequences), and the PBS group (untreated blank control). The culture medium system consisted of RPMI 1640 medium (800 μL/well), penicillin-streptomycin (2 μL/well), amphotericin B (1 μL/well), and RNasin (1 μL/well). RNAi was performed using a soaking method with liposome-mediated delivery. Following the transfection protocols established for *C. elegans* (44, 45) and cultured cells, 100 μL/well of OPTI-MEM^®^ (1X) medium was mixed with 4.5 μL/well of Lipofectamine^TM^ RNAiMAX (Thermo Fisher Scientific, MA, USA) and incubated for 5 min to prepare solution A. In parallel, 100 μL/well of OPTI-MEM^®^ (1X) medium was combined with 5 μL/well of siRNA in another centrifuge tube to prepare solution B. Under the same conditions, NC siRNA and PBS were included as negative and untreated controls, respectively. Solutions A and B were then combined, mixed thoroughly, and incubated for 15 min. The prepared transfection mixture was added to a 24-well cell culture plate containing the xL3 and culture medium, and the plate was incubated at 37°C with 5% CO_2_ for 3 days.

**Table 1:** siRNA sequence of GCY-12 gene.

**ID**	**Sense (5^′^-3^′^)**	**Antisense (5^′^-3^′^)**	**Position**
GCY-12-siRNA1	GGAACAGACAAAGGACAUUTT	AAUGUCCUUUGUCUGUUCCTT	870
GCY-12-siRNA2	GCGUCCGAUUCCUCAUCAUTT	AUGAUGAGGAAUCGGACGCTT	235
GCY-12-siRNA3	GCGCUAUGUCGAGAACAUATT	UAUGUUCUCGACAUAGCGCTT	661
NC	CUACCUGUUCCAUGGCCAATT	UUGGCCAUGGAACAGGUAGTT	

The RNAi system for *H. contortus* eggs followed the same protocol as for xL3. However, incubation at 37°C induced egg development. While this did not affect egg viability, it prevented further development in the egg stage. Further optimization of the incubation temperature for *H. contortus* eggs is required.

### Optimization of RNAi temperature of *H. contortus* eggs

To determine the optimal temperature for GCY-12 gene interference in *H. contortus* eggs, a 24-well cell culture plate was incubated at different temperatures for RNAi assays, following the same interference system as used for xL3. Five temperature gradients (4°C, 8°C, 12°C, 16°C, and 20°C) were tested. After RNAi treatment, the eggs were incubated for 3 days, and their development was observed under an inverted microscope. If all eggs remained at the undeveloped stage without further progression, and upon transfer to 27°C, the hatching rate in all negative control wells exceeded 70%, the results were considered valid. The optimal interference temperature was determined based on these criteria and subsequently used for RNAi assays in eggs.

### Extraction of total RNA and synthesis of cDNA

Total RNA was extracted separately from *H. contortus* L3 and eggs after washing with RNase- free water. RNA extraction was performed using the RNAiso Easy reagent kit (TaKaRa, Code No. TCH020) following the manufacturer's protocol. Briefly, 500 μL of RNAiso Easy reagent was added to the samples, which were then homogenized thoroughly. The mixture was incubated at room temperature for 5 min, followed by the addition of 200 μL of solution H. The lysate was mixed by inversion and centrifuged at 12,000 × g for 15 min. The supernatant was collected, and an equal volume (650 μL) of isopropanol was added. The mixture was incubated at room temperature for 10 min and centrifuged at 12,000 × g for 10 min. The precipitate was washed with 75% ethanol, centrifuged at 8,000 × g for 3 min, and the washing step was repeated. The supernatant was discarded, and pellet was air-dried for 5 min at room temperature before dissolving in 20 μL of RNase-free water. RNA concentration and purity were determined using a NanoDrop 2000 spectrophotometer. For cDNA synthesis, total RNA was first diluted to the same concentration. Genomic DNA was removed using the PrimeScript^TM^ RT reagent kit with gDNA eraser (Perfect real time) (TaKaRa, Code No. RR048A). The reaction system consisted of 2 μL of 5 × gDNA Eraser Buffer, 1 μL of gDNA eraser, an appropriate amount of RNA, and RNase-free water, adjusted to a final volume of 10 μL. The reaction was incubated at 42°C for 2 min, followed by a hold at 4°C. cDNA synthesis was then carried out using 10 μL of the reaction solution, 1 μL of PrimeScript RT Enzyme Mix I, 4 μL of primer mix, 24 μL of 5 × PrimeScript buffer, and 1 μL of RNase-free water. The reaction conditions were as follows: 37°C for 15min, 85°C for 5 s, and 4°C for 10 min. The synthesized cDNA was stored at −20°C for qRT-PCR.

### Detection of gene expression by qRT-PCR

Following siRNA interference, qRT-PCR was performed to assess gene silencing efficiency by measuring the mRNA expression levels of the target gene (46). The silencing efficiency (%) was calculated as follows:


$$\begin{array}{l} \text{Silencing efficiency }=\;\left(1\;-\text{ Relative expression}\right)\;\times \;100\% \end{array}$$

The target gene expression were measured by qRT-PCR with β-tubulin serving as the internal reference gene. qRT-PCR primers for the differentially expressed candidate gene GCY-12 and β-tubulin were designed by oligo7 software and synthesized by Beijing TSINGKE Biotech Co. Ltd. The primer sequences are shown in Table 2. qRT-PCR was performed using the TB Green^®^ Premix Ex Taq^TM^ II (Tli RNaseH Plus) kit (TaKaRa, Code No. RR820A) according to the manufacturer's protocol. Reactions were conducted on an Applied Biosystems QuantStudio 7 Flex Real-Time PCR machine (Thermo Fisher Scientific, MA, USA) in a total volume of 20 μL, consisting of 10 μL TB Green premix EX Taq II, 1 μL diluted cDNA, 7.4 μL RNase-free water, and 0.8 μL of each 10 μM primer. The relative expression levels of target genes were determined using the 2^−ΔΔCt^ method (47). Each experiment included three biological replicates, with three technical replicates per gene. Statistical significance was determined by using Student's t-test in GraphPad Prism (Version 8.0.2). Quantitative data are presented as the means ± standard deviation (SD), and statistical significance was defined as *P* < 0.05 (ns, not significant; ^*^P < 0.05; ^**^P < 0.01; ^***^P < 0.001; ^****^P < 0.0001).

**Table 2:** Primer sequences used for qRT-PCR analysis.

**mRNA ID**	**Primer (5^′^to 3^′^)**
β-tubulin	TGCTATGTTCCGTGGTCGTATG
	CGGCAGTCTTAACGTTGTTTGG
GCY-12	TGGAACAGACAAAGGACATTACG
	GGCATCGAGGTAGATCAGAAGA

### Egg hatching inhibition test after RNAi

EHA, one of the anthelmintic resistance detection methods recommended by the World Association for the Advancement of Veterinary Parasitology (WAAVP) (48), was conducted to evaluate *H. contortus* resistance to albendazole. This assay measures the ability of albendazole to inhibit the embryonation and hatching of freshly collected nematode eggs to determine the EC_50_. A sample is considered resistant if the EC_50_ of ABZ is equal to or >0.1 μg/mL. After RNAi treatment, the eggs were washed three times with normal saline. Each well in a 24-well cell culture plate contained a total volume of 400 μL, consisting of: 80 μL of RPMI 1640 culture medium, 120 μL amphotericin B (final concentration of 200 μg/mL), 120 μL of penicillin-streptomycin (final concentration of 20 μg/mL), 40 μL of washed eggs (about 100 eggs/well), 40 μL albendazole working solution at concentrations of 1 μg/mL, 0.5 μg/mL, 0.25 μg/mL, 0.125 μg/mL, 0.0625 μg/mL, and 0.03125 μg/mL. For the negative control, 40 μL of 0.5% dimethyl sulfoxide (DMSO) (Solarbio, Beijing, China) was used instead of the albendazole working solution. Each drug concentration and control group were tested in triplicate. The plates were incubated at 27°C under constant temperature and humidity conditions for 48 h.

### Statistics and analysis of EHAs

After performing the EHAs (49), the samples were examined under the inverted microscope to determine the egg hatching rate, adjusted egg hatching rate, and corrected egg hatching inhibition rate. Data analysis was conducted using the probability unit method. A logarithmic transformation of the six albendazole concentrations was plotted on the x-axis (natural logarithm, LN), while the probability unit (NORM.S.INV corrected egg hatching inhibition rate + 5) was plotted on the y-axis using Microsoft Excel. A linear regression equation was established, and the EC_50_ was calculated based on the regression equation. The formulas used for calculations were as follows:

Egg hatching rate = Number of hatched larvae/(Number of hatched larvae + Number of dead eggs + Number of remaining embryonic eggs) × 100%
Adjusted egg hatching rate = Egg hatching rate of the experimental group/Egg hatching rate of the DMSO control group × 100%;
Corrected egg hatching inhibition rate = 1-Adjusted egg hatching rate;
EC_50_ = exp ((5b)/a).

## Results

### Differentially expressed genes in dauer stage-related signaling pathways

**Figure 1:**
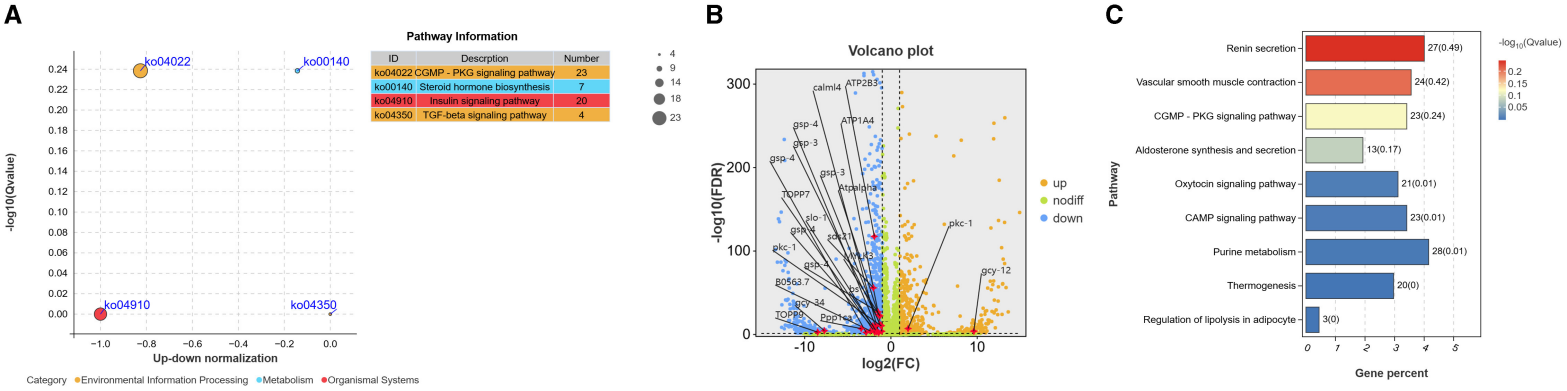
**(A)** Bubble map of differentially expressed genes in cGMP, ILS, TGF-β and hormone-like signaling pathways. **(B)** Volcano plot of significantly differentially expressed genes enriched in the cGMP signaling pathway. Orange dots: up-regulated expression; blue dots: down-regulated expression; green dots: no significant difference. **(C)** Pathways significantly enriched by GCY-12 genes.

In the comparison of sensitive and resistant strains of *H. contortus*, it was found that 23 of the 54 differentially expressed genes related to dauer stage were significantly enriched in cGMP signaling pathway (Figure 1A), accounting for the largest proportion among the four related pathways. These genes include HCON_00043720 (GCY-12), HCON_00148140 (TOPP9), HCON_00086810 (GCY-34), HCON_00162870 (PPP1CA) and HCON_00112620 (GSP-4). Among them, there were 2 up-regulated expression genes and 21 down-regulated expression genes (Figure 1B). In addition, there were 20 differentially expressed genes (20 down-regulated expression) were enriched in Insulin signaling pathway, 7 differentially expressed genes (4 down-regulated expression and 3 up-regulated expression) enriched in Steroid hormone biosynthesis signaling pathway, 4 differentially expressed genes (2 down-regulated expression and 2 up-regulated expression) in TGF-β signaling pathway. The expression of all the genes enriched in cGMP, ILS, TGF-β and hormone-like signaling pathways is shown in Supplementary Table S1. Further analysis revealed that the GCY-12 gene was significantly up-regulated expression between resistant and sensitive strains (log_2_Foldchange = 9.61, *p* < 0.05). Functional enrichment analysis showed that the gene was also significantly enriched in other eight signaling pathways, including cAMP signaling pathway, Aldosterone synthesis and secretion, Renin secretion, and Purine metabolism (Figure 1C). Based on its significant expression differences and extensive functional associations, GCY-12 was used as a candidate gene for further functional experimental verification.

### Optimization of egg interference temperature

The eggs with gene interference were cultured in biochemical incubators at 4°C, 8°C, 12°C, 16°C and 20°C for 3 days, in which the eggs had developed to the larval stage after incubation at 16°C. Therefore, this temperature condition is excluded (because the subsequent EHA requires fresh eggs to be tested, which means that the eggs should be morula stage before the experiment. When cultured at 20°C for 1 day, some eggs had developed into L1, and after 3 days, most of the larvae had reached L2, and this temperature is not available. After interfering at 4°C and 8°C for 3 days, the eggs were still mulberry eggs. After 48 h of incubation at 27°C in an incubator with albendazole, it was found that the hatching rates of negative control wells were all <70%, and the experimental data were meaningless. After gene interference at 12°C for 3 days, the eggs were in the morula stage. After adding albendazole in constant temperature and humidity incubator at 27°C for 48 h, it was found that the hatching rates of negative control holes were higher than 70% and the results were stable. Therefore, 12°C can be used as the best temperature for egg interference of *H. contortus*.

### qRT-PCR detection after RNAi

**Figure 2:**
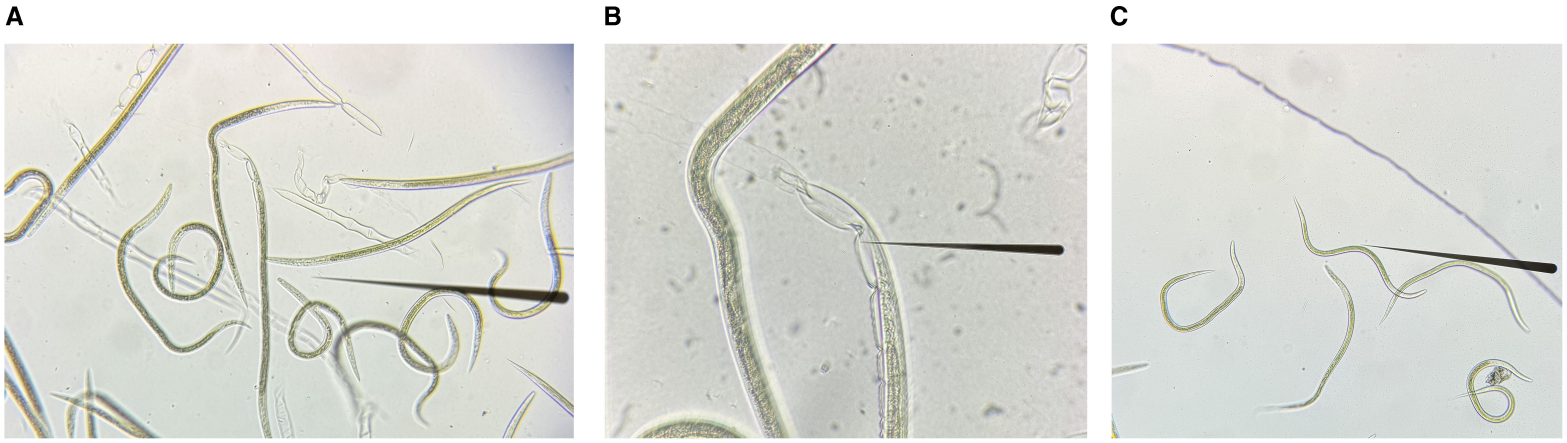
The exsheathment process in L3 of *H. contortus*. **(A)** L3 and their sheaths. **(B)** L3 undergoing exsheathment. **(C)** Post-exsheathment L3.

**Figure 3:**
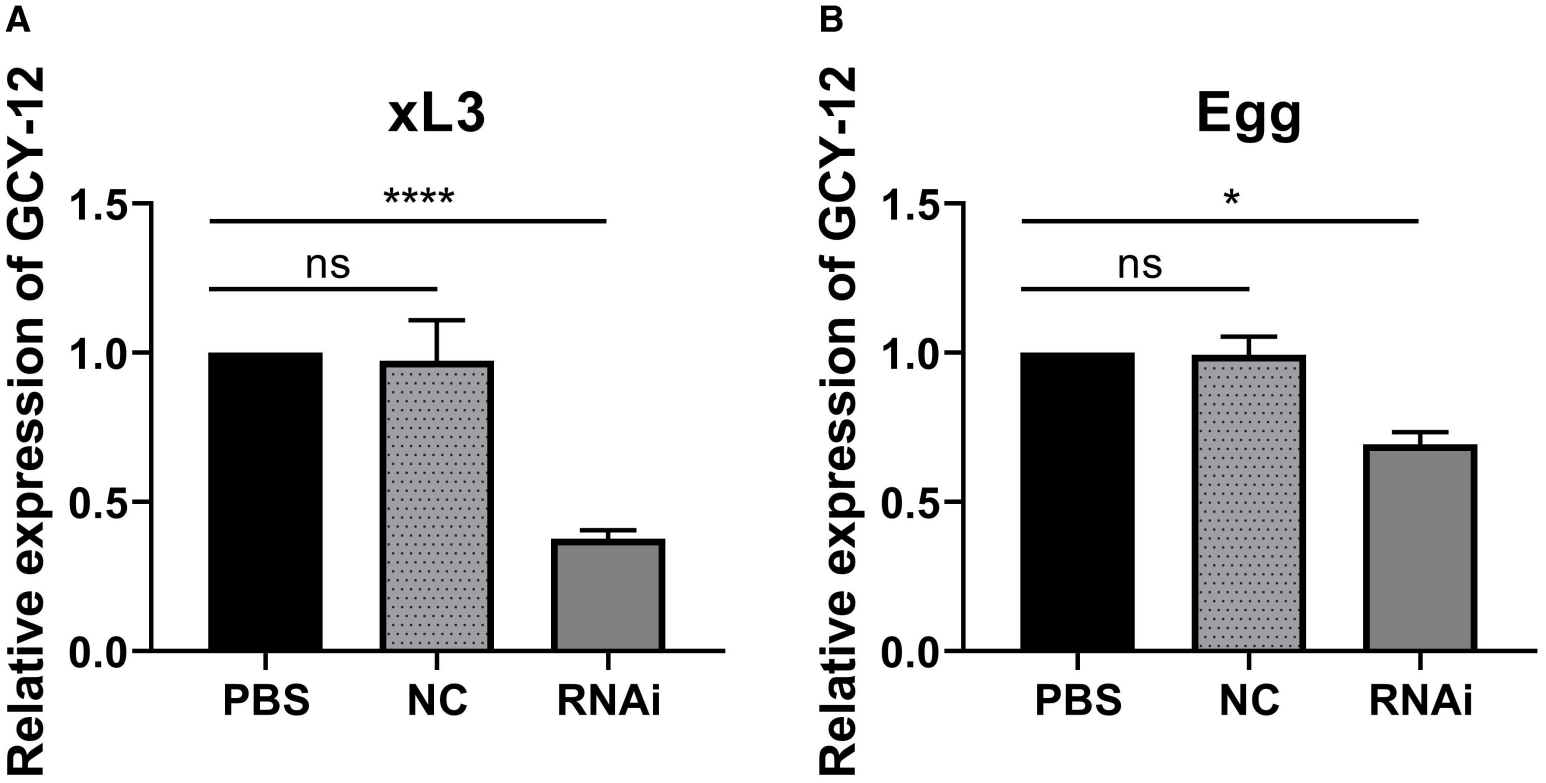
Expression of GCY-12 gene after interference by xL3 and eggs of *H. contortus*. **(A)** Expression of GCY-12 gene in xL3. **(B)** Expression of GCY-12 gene in egg. *P < 0.05. ****P < 0.0001. ns, No significant difference (*P* > 0.05).

The L3 of *H. contortus* treated with sodium hypochlorite solution are shown in Figures 2A–C. The quality of RNA extracted from xL3 and eggs after interference was tested, and the RNA quality was good (OD260/280 ratios were between 1.8 and 2.0). Subsequently, all RNA samples were diluted to the same concentration, and the gene expression was detected by qRT-PCR. β-tubulin was used as an internal reference gene to calculate the degree of silence of GCY-12 after interference (Among the three designed siRNAs, the silencing effect of GCY-12-siRNA1 designed for site 870 was significant, so this siRNA was selected as the interference sequence for subsequent experiments). The qRT-PCR results showed that the transcription level of GCY-12 was significantly reduced (a fold change of 0.36–0.41 in L3 and 0.66–0.74 in eggs). In the L3, the GCY-12 interference group showed a 59–64% reduction compared with the control group (*P* < 0.0001, Figure 3A); in the eggs, the GCY-12 interference group showed a 26–34% reduction compared with the control group (*P* < 0.05, Figure 3B), and there was no significant difference between the PBS control group and the NC control group. The expression of the target gene in the GCY-12 interference group of the eggs and the L3 GCY-12 interference group showed the same expression trend, but the expression of the target gene in the eggs was upregulated compared with that in the L3.

### Egg hatching assays after RNAi

**Figure 4:**
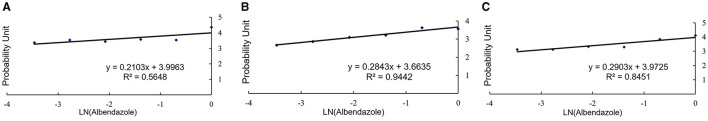
EHAs after RNAi. **(A)** PBS control group. **(B)** NC siRNA control group. **(C)** CGY-12 interference treatment group.

After the disturbance of *H. contortus* eggs, the EHAs were carried out, different concentrations of drugs were added, and the number of eggs and larvae was observed under the microscope after incubation at 27°C for 48 h, and the EC_50_ value was calculated. In the control groups, the EC_50_ values were 118.24 μg/mL for the PBS- treated group and 110.06 μg/mL for the NC-treated group. In contrast, the EC_50_ value for the CGY-12 interference group was significantly lower at 34.45 μg/mL (*P* < 0.0001). Compared with the PBS-treated group, GCY-12 interference led to a 70.86% reduction in the EC_50_ value. The results of EHA following RNAi were shown in Figures 4A–C.

## Discussion

As a widely parasitic nematode in small ruminants, *H. contortus* has caused substantial economic losses to the global livestock industry. The excessive use of anthelmintics has led to the emergence of parasite resistance, which poses a major challenge to effective parasite control. Previous studies have demonstrated that albendazole resistance in *H. contortus* is closely associated with single nucleotide mutations in the β-tubulin isotype I gene. However, accumulating evidence suggests that drug resistance is a multifactorial phenomenon involving aberrant gene expression, RNA interactions, and alterations in signal transduction pathways (50, 51).

In this study, based on transcriptome data analysis from our previous research on albendazole-sensitive and albendazole-resistant strains of *H. contortus*, we identified four dauer-related signaling pathways: cGMP, ILS, TGF-β, and hormone-like pathways, which were associated with 23, 20, 4, and 7 differentially expressed genes (DEGs), respectively. Further analysis revealed that the cGMP signaling pathway exhibited the highest proportion of DEGs, with the GCY-12 gene showing the most significant differential expression. Previous studies have established that GCY-12, a member of the receptor-type guanylate cyclase family, is expressed in multiple sensory neurons of *C. elegans* and localized in sensory cilia. It activates downstream cGMP-dependent signaling pathways, playing a crucial role in larval development and motor behavior. Additionally, GCY-12 interacts with other components of the cGMP signaling pathway to regulate sensory neuron function (52, 53). Further pathway enrichment analysis indicated that GCY-12 is also involved in eight additional signaling pathways, including the cAMP signaling pathway and renin secretion, suggesting its potential role in multiple biological processes. Despite its importance as a regulatory gene in model organisms, no studies have explored the relationship between GCY-12 and drug resistance in *H. contortus*. Therefore, investigating the role of GCY-12 in anthelmintic resistance could provide novel insights into its regulatory function in the mechanism of drug action.

RNAi technology enables the targeted silencing of specific genes and is widely used in gene function verification (54). In this study, RNAi was employed to knock down the candidate gene GCY-12 to assess its role in albendazole resistance. Our results demonstrated that RNAi-mediated silencing of GCY-12 significantly increased the sensitivity of *H. contortus* to albendazole, suggesting its potential involvement in drug response pathways. GCY-12 encodes a guanylate cyclase that participates in neuronal signaling and cellular responses to environmental stimuli, primarily through cGMP-mediated pathways. In *C. elegans*, GCY-12 has been implicated in sensory transduction and behavioral responses, suggesting a potential role in drug sensitivity regulation via neuronal or cellular signaling pathways. One possible mechanism is that GCY-12 enhances parasite resilience under adverse conditions by activating stress response mechanisms, thereby enabling *H. contortus* to withstand drug-induced damage. Silencing GCY-12 may attenuate these protective responses, rendering the parasite more susceptible to albendazole. Additionally, GCY-12 is enriched in the cGMP signaling pathway, which modulates multiple downstream effectors, including ion channels that regulate cellular osmotic balance (55). Elevated GCY-12 expression in resistant strains may alter ion channel function, thereby influencing intracellular drug concentrations. RNAi-mediated transient silencing of GCY-12 may disrupt these pathways, leading to increased intracellular accumulation of albendazole or heightened sensitivity to its cytotoxic effects. Consistent with previous studies on drug response pathways in parasitic nematodes, GCY-12 knockdown enhanced *H. contortus* sensitivity to albendazole. For instance, studies have identified several key genes involved in drug metabolism and resistance mechanisms. ABC transporters, which function as transmembrane transporters, facilitate the efflux of xenobiotics and contribute to detoxification. Xiao demonstrated that RNAi-mediated silencing of ABC transporter-related genes in the Oriental fruit fly (*Bactrocera dorsalis*) increased its susceptibility to malathion (56). Similarly, cytochrome P450 monooxygenases (P450), a family of broad-spectrum biocatalytic enzymes, play a critical role in detoxification and anthelmintic resistance. Wu reported that nine P450 genes exhibited significantly higher expression in thiamethoxam-resistant strains of the *Aphis gossypii Glover* compared to sensitive strains. RNAi-mediated inhibition of CYP6CY14 expression increased the susceptibility of resistant aphids to thiamethoxam (57). Glutathione S-transferases (GST), a widely distributed detoxification enzyme family, are also involved in insecticide resistance. In *Anopheles gambiae* strains resistant to insecticide DDT, GST-related genes exhibited increased expression at both mRNA and protein levels. Similarly, UDP-glycosyltransferases (UGTs) contribute to drug metabolism and resistance. Matoušková found that UGT368B2 expression was significantly upregulated in two benzimidazole-resistant *H. contortus* strains (IRE and WR) compared to the drug-sensitive ISE strain. Since resistant strains can more effectively inactivate benzimidazole anthelmintics through glycosylation, the constitutive overexpression of UGT368B2 may contribute to anthelmintic resistance in *H. contortus* (58). These genes are often associated with drug efflux or metabolism, reducing intracellular drug accumulation and enhancing resistance. Unlike classical resistance mechanisms, such as P-gp, which actively expel anthelmintic drugs from cells, or P450 enzymes, which facilitate drug detoxification through metabolic modification, GCY-12 appears to influence resistance through changes in its expression levels and a distinct mechanism involving cGMP signaling pathway. This suggests that resistance in *H. contortus* may result from a combination of metabolic, efflux-based, and signaling-related adaptations, broadening our understanding of anthelmintic resistance pathways. This distinction implies that GCY-12 may play a complementary rather than redundant role in the resistance network of *H. contortus*, although the precise mechanism requires further investigation.

The FECRT is the gold standard for detecting *H. contortus* drug resistance. Studies indicate that the EHA and FECRT exhibit ~90% consistency in detecting albendazole resistance (49, 59–62). The EHA is the preferred method for detecting albendazole resistance in *H. contortus*. Since albendazole exhibits no significant effect on L3, using this developmental stage for functional validation following RNAi may yield suboptimal results. Although certain genes may be associated with drug resistance, RNAi technology does not achieve complete gene silencing, limiting its effectiveness in verifying their role in resistance mechanisms. Additionally, the direct impact of RNAi on *H. contortus* eggs remains unexplored. To address this, we investigated the drug resistance of albendazole-resistant eggs following RNAi treatment. RNAi effects typically become apparent 6 h after immersion, reaching peak efficiency within 2 to 3 days, and persisting for over 72 h. Our previous research explored optimal RNAi silencing durations, revealing that GCY-12 gene expression began to decline after 1 day of interference, decreased by 61.5% on the third day, and remained stable after removing the interference solution. However, the silencing efficiency gradually diminished over time, with GCY-12 expression returning to baseline by day 12. The RNAi-mediated knockdown of GCY-12 lasted for ~11 days. Although 3 days may not represent the optimal timeframe for transcriptional downregulation, the follow-up experiments were designed based on extensive literature review and experimental feasibility assessments. Significant differences in GCY-12 gene expression were observed between albendazole-sensitive and albendazole-resistant strains. Given that GCY-12 is annotated in the cGMP signaling pathway, we selected it as an RNAi target to evaluate its role in albendazole resistance in *H. contortus*. RNAi was applied to *H. contortus* at both the L3 and egg stage, with fresh eggs exposed to interference conditions at an optimized temperature of 12°C for 3 days. Compared to the PBS control group, GCY-12 expression in L3 decreased by 59–64%, while in eggs, expression was reduced by 26–34%. This study confirms that GCY-12 is involved in albendazole resistance in *H. contortus*. However, despite the increased sensitivity of RNAi-treated eggs to albendazole, they remained resistant, supporting the hypothesis that albendazole resistance in *H. contortus* is not mediated by a single gene but rather results from the cooperative action of multiple genes. Furthermore, the RNAi effect was less pronounced in eggs than in L3, potentially due to the structural properties of the eggshell, which may limit the penetration of the interference solution. After 3 days of soaking, only a portion of the solution likely entered the eggs. Additionally, fresh eggs temporarily ceased development under these conditions but resumed development once exposed to an appropriate temperature duration. These factors suggest that the RNAi immersion method only partially influences egg-stage parasites. Although RNAi is a powerful tool for studying gene function, potential off-target effects remain a limitation. To minimize off-target effects, we performed a BLAST search to ensure siRNA sequence specificity and reduce unintended gene silencing. To enhance gene silencing specificity, we also performed qRT-PCR analysis to validate the designed siRNA sequences, ensuring the selection of siRNA sequences best suited for stable GCY-12 silencing while minimizing off-target effects. The consistent phenotypic changes observed following target gene silencing, combined with the absence of significant alterations in the control group, suggest that off-target effects did not substantially impact our findings. To further mitigate such effects, future studies may employ alternative gene-editing approaches, such as CRISPR/Cas9-mediated gene knockout, to validate these results. While our findings provide evidence for the role of GCY-12 in albendazole sensitivity, the precise molecular mechanisms remain unclear. Future research should identify downstream targets of GCY-12 through multi-omics analyses and conduct gene knockout or overexpression studies on related genes to elucidate its role in drug response pathways.

Anthelmintic resistance presents a significant challenge in livestock parasite management, particularly concerning drugs such as albendazole. Our findings indicate that the GCY-12 gene plays a role in regulating albendazole resistance, and its suppression enhances drug sensitivity. This suggests that GCY-12 and its associated signaling pathways may serve as potential targets for improving anthelmintic efficacy. By targeting GCY-12 or its related pathways, novel therapeutic strategies could be developed to restore drug sensitivity in resistant parasite populations, complementing existing treatment approaches. Furthermore, this study provides new insights into combination therapy development. Traditional albendazole resistance research has primarily focused on mutations in the β-tubulin gene as the principal resistance marker. However, our results suggest that cGMP-associated pathways may also influence drug sensitivity, highlighting the need to consider multifactorial resistance mechanisms in diagnostic screening and resistance monitoring programs. Additionally, the expression levels of GCY-12 or its downstream effectors hold potential as biomarkers for monitoring *H. contortus* resistance in pasture populations. By assessing GCY-12 expression changes, informed decisions can be made regarding drug selection and treatment regimens, ultimately improving the sustainability of parasite control strategies. Despite the potential applications of our findings, the underlying molecular interactions governing GCY-12-mediated resistance require further investigation.

## Conclusion

In this study, the system of *H. contortus* L3 and egg RNAi was established, and the temperature of interference to eggs was optimized. The GCY-12 expression levels of L3 and eggs were significantly inhibited after RNAi, and the sensitivity of *H. contortus* eggs to albendazole increased after interference.

## Data Availability

The datasets of this article are included within the manuscript and its Supplementary material. The RNA-seq raw data were submitted to the NCBI under the Accession numbers: SRR31556380 and SRR31556379.

## References

[1] Kapritchkoff RTI Okino CH Niciura SCM Bello HJS Matos RS Melito GR. Association of β-globin polymorphisms and tolerance to haemonchosis in ewes and lambs of different sheep breeds. Vet Parasitol. (2024) 328:110163. 10.1016/j.vetpar.2024.110163 38513446

[2] Maichomo MW Kagira JM Walker T. The point prevalence of gastro-intestinal parasites in calves, sheep and goats in Magadi division, south-western Kenya. Onderstepoort J Vet Res. (2004) 71:257–61. 10.4102/ojvr.v71i4.229 15732452

[3] Qamar W Alkheraije KA. Anthelmintic resistance in *Haemonchus contortus* of sheep and goats from asia–a review of *in vitro and in vivo* studies. Pak Vet J. (2023) 43:376–87. 10.29261/pakvetj/2023.088

[4] Besier RB Kahn LP Sargison ND Van Wyk JA. The pathophysiology, ecology and epidemiology of *Haemonchus contortus* infection in small ruminants. Adv Parasitol. (2016) 93:95–143. 10.1016/bs.apar.2016.02.022 27238004

[5] Wade SE Haschek WM Georgi JR. Ostertagiosis in captive bison in New York State: report of nine cases. Cornell Vet. (1979) 69:198–205. 157257

[6] Pandey VS Ndao M Kumar V. Seasonal prevalence of gastrointestinal nematodes in communal land goats from the highveld of Zimbabwe. Vet Parasitol. (1994) 51:241–8. 10.1016/0304-4017(94)90161-9 8171826

[7] KippKRRedmanEMLuksovskyJLClaussenDGilleardJSVerocaiGG. High frequency of benzimidazole resistance polymorphisms and age-class differences in trichostrongyle nematodes of ranched bison from the south-central United States. Int J Parasitol Drugs Drug Resist. (2025) 28:100594. 10.1016/j.ijpddr.2025.10059440245470

[8] Bull K Glover MJ Rose Vineer H Morgan ER. Increasing resistance to multiple anthelmintic classes in gastrointestinal nematodes on sheep farms in southwest England. Vet Rec. (2022) 190:e1531. 10.1002/vetr.1531 35338780 PMC9310741

[9] Khan T Khan W Iqbal R Maqbool A Fadladdin Y a. J, Sabtain T. Prevalence of gastrointestinal parasitic infection in cows and buffaloes in Lower Dir, Khyber Pakhtunkhwa, Pakistan. Braz J Biol. (2022) 83:e242677. 10.1590/1519-6984.242677 35137844

[10] Ali Haider A Hussain K Mares MM Abbas A Mohsin M Rehman A. *In vitro and In vivo* anthelmintic activity of *Nicotiana tabacum* against *Haemonchus placei* in Cattle. Pak Vet J. (2024) 44:745–750. 10.29261/pakvetj/2024.245

[11] Preston S Jabbar A Nowell C Joachim A Ruttkowski B Baell J. Low cost whole-organism screening of compounds for anthelmintic activity. Int J Parasitol. (2015) 45:333–43. 10.1016/j.ijpara.2015.01.007 25746136

[12] Rehman T El-Mansi AA Al-hag S Al-Shuraym LA Saeed Z Arif M. Antiparasitic activity of methanolic and ethyl acetate extracts of azadirachta indica against haemonchus contortus. Pak Vet J. (2023) 43:199–203. 10.29261/pakvetj/2023.014

[13] Velázquez-Antunez J Olivares-Perez J Olmedo-Juárez A Rojas-Hernandez S Villa-Mancera A Romero T. Biological activity of the secondary compounds of guazuma ulmifolia leaves to inhibit the hatching of eggs of haemonchus contortus. Pak Vet J. (2023) 43:55–60. 10.29261/pakvetj/2022.075

[14] Reyes-Guerrero DE Jiménez-Jacinto V Alonso-Morales RA Alonso-Díaz MÁ Maza-Lopez J Camas-Pereyra R. Assembly and analysis of *Haemonchus contortus* transcriptome as a tool for the knowledge of ivermectin resistance mechanisms. Pathogens. (2023) 12:499. 10.3390/pathogens12030499 36986421 PMC10059914

[15] Barrere V Falzon LC Shakya KP Menzies PI Peregrine AS Prichard RK. Assessment of benzimidazole resistance in *Haemonchus contortus* in sheep flocks in Ontario, Canada: comparison of detection methods for drug resistance. Vet Parasitol. (2013) 198:159–65. 10.1016/j.vetpar.2013.07.040 23993632

[16] dos Santos JML Monteiro JP Ribeiro WLC Macedo ITF Camurça-Vasconcelos ALF Vieira L da S. Identification and quantification of benzimidazole resistance polymorphisms in *Haemonchus contortus* isolated in Northeastern Brazil. Vet Parasitol. (2014) 199:160–4. 10.1016/j.vetpar.2013.11.006 24295955

[17] Reyes-Guerrero DE Higuera-Piedrahita RI Maza-Lopez J. Mendoza-de-Gives P, Camas-Pereyra R, López-Arellano ME. Analysis of P-gp genes relative expression associated to ivermectin resistance in *Haemonchus contortus* larval stages from in vitro cultures (L3 and xL3) and from gerbils (Meriones unguiculatus) (L4) as models of study. J Helminthol. (2024) 98:e19. 10.1017/S0022149X24000087 38356358

[18] Babják M Königová A Komáromyová M Kuzmina T Nosal P Várady M. Multidrug resistance in *Haemonchus contortus* in sheep - can it be overcome? J Vet Res. 67:575–581. 10.2478/jvetres-2023-0057 38130458 PMC10730552

[19] Prichard R. Anthelmintic resistance. Vet Parasitol. (1994) 54:259–68. 10.1016/0304-4017(94)90094-9 7846855

[20] Abubakar M Oneeb M Rashid M Ashraf K Chishti G Awan F. *In vitro* anthelmintic efficacy of three plant extracts against various developmental stages of Haemonchus contortus. Pak Vet J. (2024) 44:238–243. 10.29261/pakvetj/2024.174

[21] Dimunová D Matoušková P Navrátilová M Nguyen LT Ambrož M Vokrál I. Environmental circulation of the anthelmintic drug albendazole affects expression and activity of resistance-related genes in the parasitic nematode Haemonchus contortus. Sci Total Environ. (2022) 822:153527. 10.1016/j.scitotenv.2022.153527 35101480

[22] Barrère V Alvarez L Suarez G Ceballos L Moreno L Lanusse C. Relationship between increased albendazole systemic exposure and changes in single nucleotide polymorphisms on the β-tubulin isotype 1 encoding gene in Haemonchus contortus. Vet Parasitol. (2012) 186:344–9. 10.1016/j.vetpar.2011.11.068 22192770

[23] Liaqat I Omer O Rasheed AM Raza S. Preparation, characterization and *in vitro* anticancer evaluation of albendazole-loaded zinc oxide nanoparticles. Pak Vet J. (2024) 44:1338–13449. 10.29261/pakvetj/2024.282

[24] von Samson-Himmelstjerna G Walsh TK Donnan AA Carrière S Jackson F Skuce PJ. Molecular detection of benzimidazole resistance in *Haemonchus contortus* using real-time PCR and pyrosequencing. Parasitology. (2009) 136:349–58. 10.1017/S003118200800543X 19154653

[25] Tan TK Lim YAL Chua KH Chai HC Low VL Bathmanaban P. Characterization of benzimidazole resistance in Haemonchus contortus: integration of phenotypic, genotypic and proteomic approaches. Parasitol Res. (2020) 119:2851–62. 10.1007/s00436-020-06790-5 32651637

[26] Kalule F Vudriko P Nanteza A Ekiri AB Alafiatayo R Betts J. Prevalence of gastrointestinal parasites and molecular identification of beta-tubulin mutations associated with benzimidazole resistance in *Haemonchus contortus* in goats from selected districts of Uganda. Vet Parasitol Reg Stud Reports. (2023) 42:100889. 10.1016/j.vprsr.2023.100889 37321794

[27] Vokrál I Bártíková H Prchal L Stuchlíková L Skálová L Szotáková B. The metabolism of flubendazole and the activities of selected biotransformation enzymes in *Haemonchus contortus* strains susceptible and resistant to anthelmintics. Parasitology. (2012) 139:1309–16. 10.1017/S0031182012000595 22717022

[28] Stuchlíková LR Matoušková P Vokrál I Lamka J Szotáková B Sečkarová A. Metabolism of albendazole, ricobendazole and flubendazole in *Haemonchus contortus* adults: Sex differences, resistance-related differences and the identification of new metabolites. Int J Parasitol Drugs Drug Resist. (2018) 8:50–8. 10.1016/j.ijpddr.2018.01.005 29414106 PMC6114105

[29] Xie G Shao Z. SPP-5 affects larval arrest via insulin signaling pathway in Caenorhabditis elegans. J Mol Histol. (2024) 55:491–502. 10.1007/s10735-024-10205-5 38869752

[30] Krishnan H Ahmed S Hubbard SR Miller WT. Catalytic activities of wild-type C. elegans DAF-2 kinase and dauer-associated mutants. FEBS J. (2024) 10.1111/febs.17303 39428852 PMC11705002

[31] Hofmann F. The cGMP system: components and function. Biol Chem. (2020) 401:447–69. 10.1515/hsz-2019-0386 31747372

[32] Li F Lok JB Gasser RB Korhonen PK Sandeman MR Shi D. Hc-daf-2 encodes an insulin-like receptor kinase in the barber's pole worm, Haemonchus contortus, and restores partial dauer regulation. Int J Parasitol. (2014) 44:485–96. 10.1016/j.ijpara.2014.03.005 24727120 PMC4516220

[33] Hu M Lok JB Ranjit N Massey HC Sternberg PW Gasser RB. Structural and functional characterisation of the fork head transcription factor-encoding gene, Hc-daf-16, from the parasitic nematode *Haemonchus contortus* (Strongylida). Int J Parasitol. (2010) 40:405–15. 10.1016/j.ijpara.2009.09.005 19796644 PMC2853935

[34] Hotez P Hawdon J Schad GA. Hookworm larval infectivity, arrest and amphiparatenesis: the Caenorhabditis elegans Daf-c paradigm. Parasitol Today. (1993) 9:23–6. 10.1016/0169-4758(93)90159-D 15463660

[35] Khan M Maryam A Mehmood T Zhang Y Ma T. Enhancing activity of anticancer drugs in multidrug resistant tumors by modulating P-Glycoprotein through dietary nutraceuticals. Asian Pac J Cancer Prev. (2015) 16:6831–9. 10.7314/APJCP.2015.16.16.6831 26514453

[36] Liu Y Luo X Li J Wang P Teng B Wang R. Using feeding and motility patterns for ivermectin resistance detecting in *Haemonchus contortus* larvae. Exp Parasitol. (2022) 238:108230. 10.1016/j.exppara.2022.108230 35151652

[37] Gray JM Hill JJ Bargmann CI. A circuit for navigation in Caenorhabditis elegans. Proc Natl Acad Sci U S A. (2005) 102:3184–91. 10.1073/pnas.0409009101 15689400 PMC546636

[38] Persson A Gross E Laurent P Busch KE Bretes H de Bono M. Natural variation in a neural globin tunes oxygen sensing in wild Caenorhabditis elegans. Nature. (2009) 458:1030–3. 10.1038/nature07820 19262507

[39] Chaubey AH Sojka SE Onukwufor JO Ezak MJ Vandermeulen MD Bowitch A. The Caenorhabditis elegans innexin INX-20 regulates nociceptive behavioral sensitivity. Genetics. (2023) 223:iyad017. 10.1093/genetics/iyad017 36753530 PMC10319955

[40] Liu Y Jiang J-J Du S-Y Mu L-S Fan J-J Hu J-C. Artemisinins ameliorate polycystic ovarian syndrome by mediating LONP1-CYP11A1 interaction. Science. (2024) 384:eadk5382. 10.1126/science.adk5382 38870290

[41] Wu T Dong J Fu J Kuang Y Chen B Gu H. The mechanism of acentrosomal spindle assembly in human oocytes. Science. (2022) 378:eabq7361. 10.1126/science.abq7361 36395215

[42] Fang H Sun Q Zhou J Zhang H Song Q Zhang H. m6A methylation reader IGF2BP2 activates endothelial cells to promote angiogenesis and metastasis of lung adenocarcinoma. Mol Cancer. (2023) 22:99. 10.1186/s12943-023-01791-1 37353784 PMC10288689

[43] Opitz B Püschel A Schmeck B Hocke AC Rosseau S Hammerschmidt S. Nucleotide-binding oligomerization domain proteins are innate immune receptors for internalized Streptococcus pneumoniae. J Biol Chem. (2004) 279:36426–32. 10.1074/jbc.M403861200 15215247

[44] He L Liu H Zhang B-Y Li F-F Di W-D Wang C-Q. A daf-7-related TGF-β ligand (Hc-tgh-2) shows important regulations on the development of Haemonchus contortus. Parasit Vectors. (2020) 13:326. 10.1186/s13071-020-04196-x 32586367 PMC7318536

[45] Zheng J Yan X Lu T Song W Li Y Liang J. CircFOXK2 promotes hepatocellular carcinoma progression and leads to a poor clinical prognosis via regulating the Warburg effect. J Exp Clin Cancer Res. (2023) 42:63. 10.1186/s13046-023-02624-1 36922872 PMC10018916

[46] Schmittgen TD Livak KJ. Analyzing real-time PCR data by the comparative C(T) method. Nat Protoc. (2008) 3:1101–8. 10.1038/nprot.2008.73 18546601

[47] Livak KJ Schmittgen TD. Analysis of relative gene expression data using real-time quantitative PCR and the 2^−ΔΔCT^ method. Methods. (2001) 25:402–8. 10.1006/meth.2001.1262 11846609

[48] Coles GC Bauer C Borgsteede FH Geerts S Klei TR Taylor MA Waller PJ. World association for the advancement of veterinary parasitology (WAAVP) methods for the detection of anthelmintic resistance in nematodes of veterinary importance. Vet Parasitol. (1992) 44:35–44. 10.1016/0304-4017(92)90141-U 1441190

[49] Maingi N Bjørn H Dangolla A. The relationship between faecal egg count reduction and the lethal dose 50% in the egg hatch assay and larval development assay. Vet Parasitol. (1998) 77:133–45. 10.1016/S0304-4017(97)00222-7 9746283

[50] Wen H Zhang Y Yang L Wang W Liu C. Study of the drug resistance function of ivermectin-resistance-related miRNAs in Haemonchus contortus. Acta Parasitol. (2025) 70:7. 10.1007/s11686-024-00964-2 39776274

[51] Kotze AC Gilleard JS Doyle SR Prichard RK. Challenges and opportunities for the adoption of molecular diagnostics for anthelmintic resistance. Int J Parasitol Drugs Drug Resist. (2020) 14:264–73. 10.1016/j.ijpddr.2020.11.005 33307336 PMC7726450

[52] Fujiwara M Hino T Miyamoto R Inada H Mori I Koga M. The importance of cgmp signaling in sensory cilia for body size regulation in Caenorhabditis elegans. Genetics. (2015) 201:1497–510. 10.1534/genetics.115.177543 26434723 PMC4676540

[53] Fujiwara M Teramoto T Ishihara T Ohshima Y McIntire SL. A novel zf-MYND protein, CHB-3, mediates guanylyl cyclase localization to sensory cilia and controls body size of Caenorhabditis elegans. PLoS Genet. (2010) 6:e1001211. 10.1371/journal.pgen.1001211 21124861 PMC2991246

[54] Chen D Li PW-L Goldstein BA Cai W Thomas EL Chen F. Germline signaling mediates the synergistically prolonged longevity produced by double mutations in daf-2 and rsks-1 in C. elegans Cell Rep. (2013) 5:1600–10. 10.1016/j.celrep.2013.11.018 24332851 PMC3904953

[55] Friebe A Sandner P Schmidtko A. cGMP: a unique 2nd messenger molecule - recent developments in cGMP research and development. Naunyn Schmiedebergs Arch Pharmacol. (2020) 393:287–302. 10.1007/s00210-019-01779-z 31853617 PMC7260148

[56] Xiao L-F Zhang W Jing T-X Zhang M-Y Miao Z-Q Wei D-D. Genome-wide identification, phylogenetic analysis, and expression profiles of ATP-binding cassette transporter genes in the oriental fruit fly, Bactrocera dorsalis (Hendel) (Diptera: Tephritidae). Comp Biochem Physiol Part D Genomics Proteomics. (2018) 25:1–8. 10.1016/j.cbd.2017.10.001 29121518

[57] Wu Y Xu H Pan Y Gao X Xi J Zhang J. Expression profile changes of cytochrome P450 genes between thiamethoxam susceptible and resistant strains of Aphis gossypii Glover. Pestic Biochem Physiol. (2018) 149:1–7. 10.1016/j.pestbp.2018.05.007 30033005

[58] Matoušková P Lecová L Laing R Dimunová D Vogel H Raisová Stuchlíková L. UDP-glycosyltransferase family in Haemonchus contortus: phylogenetic analysis, constitutive expression, sex-differences and resistance-related differences. Int J Parasitol Drugs Drug Resist. (2018) 8:420–9. 10.1016/j.ijpddr.2018.09.005 30293057 PMC6174829

[59] Coles GC Jackson F Pomroy WE Prichard RK von Samson-Himmelstjerna G Silvestre A. The detection of anthelmintic resistance in nematodes of veterinary importance. Vet Parasitol. (2006) 136:167–85. 10.1016/j.vetpar.2005.11.019 16427201

[60] Powers KG Wood IB Eckert J Gibson T Smith HJ. World Association for the Advancement of Veterinary Parasitology (WAAVP) guidelines for evaluating the efficacy of anthelmintics in ruminants (bovine and ovine). Vet Parasitol. (1982) 10:265–84. 10.1016/0304-4017(82)90078-4 6753316

[61] Wood IB Amaral NK Bairden K Duncan JL Kassai T Malone JB. World Association for the Advancement of Veterinary Parasitology (WAAVP) second edition of guidelines for evaluating the efficacy of anthelmintics in ruminants (bovine, ovine, caprine). Vet Parasitol. (1995) 58:181–213. 10.1016/0304-4017(95)00806-2 7571325

[62] Malone JB Soulsby EJL Roncalli R. History of the world association for the advancement of veterinary parasitology (WAAVP). Vet Parasitol. (2004) 125:3–18. 10.1016/j.vetpar.2004.05.002 15476964

